# Psychosocial stress accompanied by an unhealthy eating behavior is associated with abdominal obesity in Korean adults: A community-based prospective cohort study

**DOI:** 10.3389/fnut.2022.949012

**Published:** 2022-09-30

**Authors:** Minji Kim, Yangha Kim

**Affiliations:** ^1^Department of Nutritional Science and Food Management, Ewha Womans University, Seoul, South Korea; ^2^Graduate Program in System Health Science and Engineering, Ewha Womans University, Seoul, South Korea

**Keywords:** psychological stress, obesity, non-communicable disease, dietary quality, dietary variety score, longitudinal study, gender stratification

## Abstract

Psychosocial stress is recognized as a potential modulator of eating behavior. Psychosocial stress also constitutes an independent risk factor for the development of non-communicable diseases. This study examined the gender-stratified associations between perceived stress, eating behavior, and abdominal obesity in 4,411 adults aged 40–69 years during a 10-year follow-up of the Korean Genome and Epidemiology Study (KoGES). Psychosocial stress was evaluated using the Psychosocial Wellbeing Index Short Form (PWI-SF), and eating behavior was analyzed with a focus on the dietary variety score (DVS). The Cox's proportional hazard model was used to examine the risk of abdominal obesity according to stress levels. Higher stress levels were associated with lower DVS in women. Lower DVS scores were positively associated with the consumption of grains and refined grains but was negatively associated with the consumption of fruits. The DVS was not significantly associated with stress levels among men. Prospectively, the highest tertile of grains and refined grains consumption showed an increased risk of abdominal obesity compared to the lowest tertile in women (HR: 1.36, 95% CI: 1.04–1.78, *p* < 0.05; HR: 1.28, 95% CI: 1.03–1.59, *p* < 0.05, respectively). By contrast, in all participants, the highest tertile of fruits consumption decreased the risk of abdominal obesity compared to the lowest tertile (men, HR: 0.56, 95% CI: 0.45–0.70, *p* < 0.01; women, HR: 0.51, 95% CI: 0.40–0.65, *p* < 0.01). Furthermore, high stress levels showed a borderline significant association with the risk of abdominal obesity only in women (HR: 1.27, 95% CI: 1.00–1.59, *p* < 0.05). These findings suggested that psychosocial stress might contribute to abdominal obesity by interacting with eating behavior represented by a low DVS. The approach to consume a diet with a high DVS might help decrease the risk of abdominal obesity among people in stressful environments.

## Introduction

Psychosocial stress, arising from the workplace or socioeconomic disadvantage and discrimination, is known to affect health outcomes through biological and behavioral changes (1). Stress-induced modification of eating behaviors may be particularly important in understanding various health outcomes. Stress appears to alter overall eating in two contrasting ways (2, 3). When individuals experience chronic stress, they may increase their food intake in response to stress; however, there is also support of either no changes in eating behavior or a reduction of food intake in response to stress (4–6). Moreover, the situational changes in stress, such as any noxious event in one's environment that could be appraised as threatening, risky or harmful, might also evoke change in eating behaviors (3). Little is known on what determines the directional changes in eating behavior following stress, though it has been suggested that the hypothalamic pituitary adrenal (HPA) axis is implicated and the eating-stress behavior relationship in those who experience chronic stress (7). The hyperactivation of the HPA axis, accompanied by increased secretion of cortisol, may entice people to consume energy-dense and hyperpalatable foods, such as those high in sugar and fat, which may then increase the risk of obesity or becoming overweight (8).

It is reported that abdominal obesity accompanied by an increase in intra-abdominal fat and waist circumference (WC) (9) is a primary risk factor for the development of metabolic disorders, such as cardiovascular disease, type 2 diabetes, metabolic syndrome, and some types of cancer (10, 11). The prevalence of abdominal obesity is rapidly rising worldwide. In the United States of America, the estimated prevalence of abdominal obesity increased from 59% in 2003–2004 to 64% in 2013–2014 in men and from 40 to 44% in women (12). In addition, a national survey in Korea reported that the prevalence of abdominal obesity increased from 19.0% in 2009 to 23.8% in 2018 (13). The modifiable lifestyle factors associated with abdominal obesity include stress levels, sedentary patterns, and unhealthy eating behavior (9, 14).

Eating behavior is a broad term that encompasses food choice and eating motives, feeding practices, dieting, and eating-related problems (15). Healthy eating behaviors have been identified as eating nutrient-balanced meals and a variety of foods (16). Dietary variety is regarded as an integral component of healthy eating behavior (17). The dietary variety score (DVS) may be an indicator for assessing eating behavior by counting the total number of different food items consumed over a period of time (18). A low DVS was intimately related to increased energy ratios of carbohydrates and grains, as well as nutritionally imbalanced meals (19). When chronically stressed, people tend to engage in unhealthy eating behaviors.

The effect of perceived stress on eating behaviors is thought to differ between men and women. Prior research in the general population has reported gender differences in emotional eating, which is occurring in the presence of negative emotions (20). Women are more likely to change their normal eating behaviors when experiencing stress compared to men (21, 22).

According to a 6.5-year follow-up in a Dutch population of middle-aged and older adults, the experience of stressful life events was associated with an increased incidence of abdominal obesity (23). A meta-analysis showed that the risk of adiposity was increased by about 25% due to psychosocial stress (24). However, to our knowledge, no prospective study has investigated whether stress may modify eating behaviors, which then may consequently contribute to the risk of abdominal obesity. Therefore, we aimed to investigate the associations between perceived stress, eating behavior, and abdominal obesity in middle-aged and older adults stratified by gender, using data from the Korean Genome and Epidemiology Study (KoGES), a large community-based cohort study. We hypothesized that stress accompanied by an unhealthy eating behavior may be associated with an increased risk of abdominal obesity. Moreover, the direction and magnitude of this association may differ by gender.

## Materials and methods

### Study population

**Figure 1:**
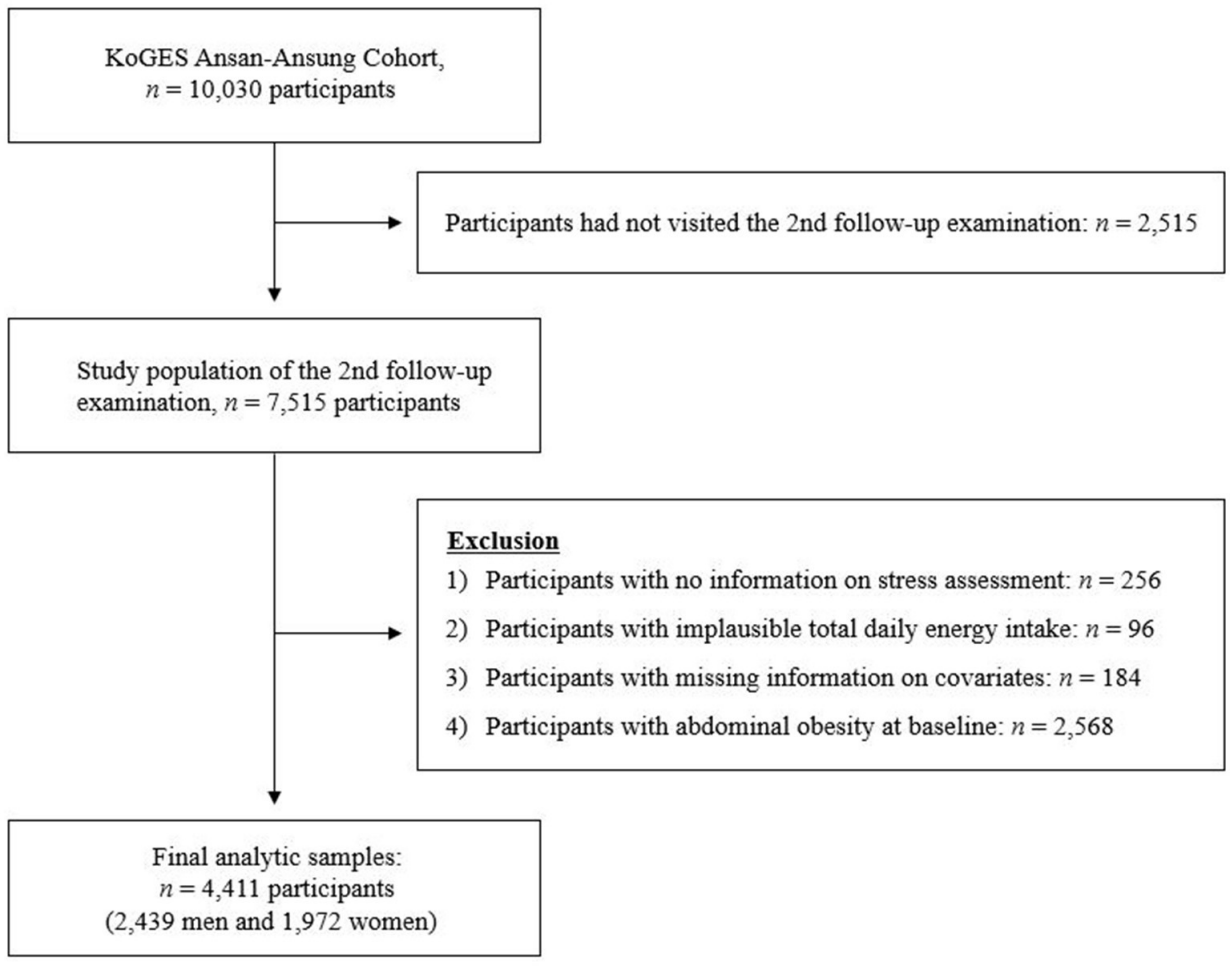
Flow diagram of the study population.

We used data from a prospective population-based Ansan-Ansung cohort study, part of the KoGES, to examine the risk and burden of chronic disease among the general Korean population. Detailed information on the study design and aims of the KoGES has been previously reported (25). In brief, 10,030 participants aged 40–69 years were recruited from the Ansan (urban) and Ansung (rural) areas, and follow-up examinations were conducted biennially. The second follow-up examination provided information on stress levels, so our analysis used this data as the baseline. Data from the baseline (2005–2006) to the seventh examination (2015–2016) were used for the current study. Among the 7,515 participants, we excluded participants who reported implausible total daily energy intake (<500 or >4,000 kcal/day, *n* = 96), those who did not respond to the stress assessment (*n* = 256), and those with missing information on covariates (*n* = 184). An additional 2,568 participants with abdominal obesity at baseline were excluded. Finally, 4,411 participants (2,439 men and 1,972 women) were analyzed (Figure 1). The study was approved by the Institutional Review Board of Ewha Womans University (2021-0316, October 2021).

### Definition of abdominal obesity

WC was measured at each follow-up examination. Abdominal obesity was defined as a WC ≥ 90 cm in men and ≥ 85 cm in women, in accordance with the definition of the Korean Society for the Study of Obesity (KSSO) (26).

### Assessment of psychosocial stress

At baseline, the participants' stress levels were assessed using the Psychosocial Wellbeing Index Short Form (PWI-SF) developed by Chang (27), which was based on the general health questionnaire devised by Goldberg (28). The validity of the PWI-SF has been previously demonstrated (27). The PWI-SF consists of 18 items: social performance and self-confidence (eight items), depression (three items), sleep disturbances and anxiety (three items), and overall wellbeing and vitality (four items). Each item ranges from “strongly disagree” (0) to “strongly agree” (3) based on a 4-point Likert scale, and total PWI score is the sum of each subscale. A higher PWI-SF score reflects a higher level of psychosocial stress.

### Assessment of food consumption and eating behavior

The dietary intake information was collected using the semi-quantitative food frequency questionnaire (FFQ) developed for the KoGES (29). This FFQ consisted of 106 food items. Food items were classified into 8 groups based on the previous study (30). We modified Leila Azadbakht's method adding highly palatable foods category: grains, refined grains, vegetables, fruits, dairy, meat, fast foods, and highly palatable foods (Supplementary Table 1).

Food consumption was measured once, at baseline of the study, concerning the individual's dietary intake over the past year. Participants were asked to report their average food frequency (on a 9-point scale of “almost none,” “once a month,” “twice or three times a month,” “once or twice a week,” “twice or three times a week,” “five or six times a week,” “once a day,” “twice a day,” and “three times a day”) and the average portion size (on a 3-point scale of “0.5 times the reference,” “reference,” and “1.5–2.0 times the reference”) for each food item for 1 year. The duration of the seasonal variety of fruit consumption was divided into four categories (3, 6, 9, and 12 months). The validation and reproducibility of the FFQ are described in detail (29).

Eating behavior was evaluated based on the DVS, originally devised by Elizabeth Randall et al. (31). In this study, we measured DVS modified by Choi et al. (18), counting the food items consumed at least once per month. Specifically, the food items consumed were counted as 1 point except “almost none,” based on the reported frequency from the FFQ. Foods consumed multiple times during the period were counted only once. In addition, the foods containing the same ingredients, such as pork roast and steamed pork, were considered as one food. Each time another food item was consumed, the DVS increased by 1 point.

### Measurements

Anthropometric measurements were obtained by trained research staff at each follow-up visit. Height and body weight were measured with the participants wearing a patient gown and no shoes, and the body mass index (BMI) was calculated as body weight (kg) divided by the square of height (m^2^). The WC (cm) was measured at the thinnest point between the lower rib and the iliac crest, and the average of three repeated measurements was used in this study. Blood pressure (BP) was measured in both arms using a mercury sphygmomanometer (W.A Baum Co. Inc., Copiague, NY, USA) after resting for at least 5 min. This study used the average value of repeated measurements to define systolic BP and diastolic BP.

### Covariates

The demographic characteristics, socioeconomic status, and lifestyle factors of the participants were surveyed at baseline. Covariates included age, BMI, marital status (others, married), monthly household income (<3 million KRW, ≥3 million KRW), education level (others, ≥college), alcohol consumption (never, former, current), smoking status (never, former, current), and physical activity (<30 min/day, ≥30 min/day).

### Statistical analysis

Continuous variables are expressed as mean and standard error (SE), and categorical variables are expressed as numbers and percentages. The generalized linear model and the Chi-square test were used to determine the differences in means and distribution of general characteristics and to test the linear trends according to stress levels. For adjustment in the multivariable model, potential confounders from the previously published scientific literature were taken into account (14, 32, 33) with stepwise regression procedures, such as age, BMI, marital status, monthly household income, education level, alcohol consumption, smoking status, physical activity and total energy intake. The multivariable Cox proportional hazard model was used to assess the hazard ratios (HRs) and 95% confidence intervals (CIs) for the risk of abdominal obesity according to stress levels during the follow-up. Data analyses were performed with SAS software, version 9.4 (SAS Institute, Cary, NC, USA). Statistical significance was considered at *P* < 0.05. We stratified the analysis according to gender, as previous research reported that gender influences the relation between stress and eating behaviors (20–22).

## Results

### Baseline characteristics

Table 1 describes the characteristics of the study population according to tertiles of stress level at baseline. Compared to those with lower stress levels, men with higher stress levels were more likely to have lower waist circumference (WC) (*p* < 0.01). However, women did not show any difference in WC among groups. In men, participants with higher levels of stress were younger (*p* < 0.01), consumed alcohol currently (*p* < 0.01), and were less physically active (*p* < 0.01), whereas women with higher levels of stress were older (*p* < 0.05). Alcohol consumption and physical activity were not significantly different with stress levels among women. In both men and women, participants with higher levels of stress had lower BMI, had lower household income, were less educated, and were more likely to be current smokers (all *p* < 0.05).

**Table 1:** Baseline characteristics of the study population.

	**Men**				**Women**			
	**T1** **(Lowest) ^§^**	**T2** **(Intermediate)**	**T3** **(Highest)**	***P*-value**	**T1** **(Lowest)**	**T2** **(Intermediate)**	**T3** **(Highest)**	***P*-value**
	**(*n* = 976)**	**(*n* = 790)**	**(*n* = 673)**		**(*n* = 495)**	**(*n* = 720)**	**(*n* = 757)**	
PWI-SF score (median)	9.0	17.0	26.0		9.0	17.0	27.0	
Age (years)	55.9 ± 0.3	54.5 ± 0.3	54.8 ± 0.3	0.0013	53.8 ± 0.4	53.2 ± 0.3	54.4 ± 0.3	0.0269
Height (cm)	166.4 ± 0.2	166.0 ± 0.2	166.5 ± 0.2	0.1294	154.1 ± 0.3	154.6 ± 0.2	153.6 ± 0.2	0.0015
Weight (kg)	64.9 ± 0.2	63.9 ± 0.3	63.0 ± 0.3	<0.0001	55.1 ± 0.3	55.2 ± 0.2	53.9 ± 0.3	0.0002
Waist circumference (cm)	82.2 ± 0.2	81.5 ± 0.2	80.8 ± 0.2	<0.0001	76.7 ± 0.2	76.8 ± 0.2	76.7 ± 0.2	0.9102
Body mass index (kg/m^2^)	23.4 ± 0.07	23.2 ± 0.08	22.7 ± 0.10	<0.0001	23.2 ± 0.10	23.1 ± 0.08	22.8 ± 0.09	0.0146
Systolic blood pressure (mmHg)	116.0 ± 0.5	115.2 ± 0.5	115.2 ± 0.6	0.4768	112.6 ± 0.8	111.4 ± 0.6	111.9 ± 0.6	0.4665
Diastolic blood pressure (mmHg)	77.8 ± 0.3	77.7 ± 0.4	77.7 ± 0.4	0.9632	74.0 ± 0.5	73.3 ± 0.4	74.0 ± 0.4	0.3297
**Marital status (%)**				0.3715				0.4612
Others	31 (3.2)	29 (3.7)	34 (5.0)		62 (15.5)	90 (15.5)	118 (15.6)	
Married	945 (96.8)	761 (96.3)	639 (95.0)		433 (87.5)	630 (87.5)	639 (84.4)	
Monthly household Income (≥3 million KRW, %)	350 (35.9)	290 (36.7)	202 (30.0)	0.0143	174 (35.2)	232 (32.2)	174 (23.0)	<0.0001
Education level (≥College, %)	208 (21.3)	177 (22.4)	111 (16.5)	0.0123	56 (11.3)	75 (10.4)	39 (5.2)	<0.0001
**Alcohol consumption (%)**				0.0067				0.4934
Never	200 (20.5)	171 (21.7)	103 (15.3)		353 (71.3)	520 (72.2)	525 (69.4)	
Past	97 (9.9)	57 (7.2)	64 (9.5)		5 (1.0)	14 (1.9)	14 (1.9)	
Current	679 (69.6)	562 (71.1)	506 (75.2)		137 (27.7)	186 (25.8)	218 (28.8)	
**Smoking status (%)**				<0.0001				0.0356
Never	295 (30.2)	183 (23.2)	125 (18.6)		476 (96.2)	706 (98.1)	724 (95.6)	
Past	372 (38.1)	308 (39.0)	228 (33.9)		6 (1.2)	5 (0.7)	5 (0.7)	
Current	309 (31.7)	299 (37.9)	320 (47.6)		13 (2.6)	9 (1.3)	28 (3.7)	
**Physical activity (%)**				<0.0001				0.1298
<30 min	269 (27.6)	261 (33.0)	262 (38.9)		173 (35.0)	264 (36.7)	305 (40.3)	
≥30 min	707 (72.4)	529 (67.0)	411 (61.1)		322 (65.1)	456 (63.3)	452 (59.7)	

KRW, Korean won. Values are expressed as mean (SE) or numbers (percentages). ^§^Stress levels were assessed using the Psychosocial Wellbeing Index-Short Form (PWI-SF). The P-value was calculated from the ANOVA test for continuous variables and the Chi-square test for categorical variables.

### Associations between stress levels and food consumption

The associations of stress levels with food consumption (g/1,000 kcal) are presented in Table 2. Among men, the DVS did not differ significantly according to stress levels, whereas women with higher levels of stress showed a lower DVS (*p* < 0.01). In men, the higher stress levels were associated with a higher consumption of refined grains (*p* < 0.05) and highly palatable foods (*p* < 0.05), but lower consumption of fruits (*p* < 0.01). In women, the higher stress levels were associated with a higher consumption of grains (*p* < 0.01), especially refined grains (*p* < 0.01), but lower consumption of fruits (*p* < 0.01), dairy (*p* < 0.05), and meat (*p* < 0.01).

**Table 2:** Food consumption according to stress levels.

	**Men**				**Women**			
**Food consumption (g/1,000 kcal)**	**T1** **(Lowest) ^§^**	**T2** **(Intermediate)**	**T3** **(Highest)**	***P*-trend**	**T1** **(Lowest)**	**T2** **(Intermediate)**	**T3** **(Highest)**	***P*-trend**
	**(*n* = 976)**	**(*n* = 790)**	**(*n* = 673)**		**(*n* = 495)**	**(*n* = 720)**	**(*n* = 757)**	
DVS	53.8 ± 0.43	53.6 ± 0.49	52.8 ± 0.54	0.6432	53.9 ± 0.55	54.5 ± 0.44	51.4 ± 0.48	0.0074
Grains	416.7 ± 2.47	423.4 ± 2.86	421.5 ± 3.07	0.2439	385.6 ± 4.07	399.9 ± 3.22	416.5 ± 3.32	<0.0001
Refined grains	136.8 ± 5.76	153.6 ± 6.84	163.1 ± 7.37	0.0431	68.0 ± 5.88	78.7 ± 5.25	101.4 ± 6.00	0.0006
Vegetables	144.7 ± 2.77	144.3 ± 3.19	141.8 ± 3.14	0.5551	148.7 ± 4.00	146.3 ± 3.35	145.1 ± 3.24	0.3219
Fruits	97.9 ± 2.71	89.7 ± 2.55	82.1 ± 2.66	0.0087	163.0 ± 5.17	154.4 ± 4.07	129.0 ± 3.63	<0.0001
Dairy	56.1 ± 2.07	53.2 ± 2.07	54.4 ± 2.44	0.8738	84.6 ± 3.67	77.6 ± 2.98	72.6 ± 2.68	0.0395
Meat	44.8 ± 0.89	42.9 ± 0.94	42.8 ± 1.02	0.1586	40.0 ± 1.22	37.1 ± 0.89	34.9 ± 0.93	0.0069
Fast foods	1.35 ± 0.15	1.28 ± 0.12	1.60 ± 0.20	0.2951	2.30 ± 0.29	2.06 ± 0.19	1.86 ± 0.18	0.4829
Highly palatable foods	17.1 ± 0.86	18.6 ± 1.02	21.1 ± 1.24	0.0206	15.1 ± 1.00	15.1 ± 1.05	15.2 ± 1.23	0.8612

DVS, dietary variety score. Values are expressed as mean (SE).

§ Stress levels were assessed using the Psychosocial Wellbeing Index-Short Form (PWI-SF). The P-trend was obtained through generalized linear models after adjusting for age, BMI, marital status, monthly household income, education level, alcohol consumption, smoking status and physical activity.

### Associations between DVS and food consumption

The associations of DVS with food consumption (g/1,000 kcal) are shown in Table 3. In all participants, as the DVS decreased, the consumption of grains and refined grains increased (all *p* < 0.05). By contrast, as the DVS decreased, the consumption of fruits, dairy, meat, fast foods, and highly palatable foods decreased (all *p* < 0.01). Consumption of vegetables was not significantly associated with DVS.

**Table 3:** Food consumption according to dietary variety score.

	**Men**				**Women**			
**Food consumption (g/1,000 kcal)**	**T1** **(Lowest)**	**T2** **(Intermediate)**	**T3** **(Highest)**	***P*-trend**	**T1** **(Lowest)**	**T2** **(Intermediate)**	**T3** **(Highest)**	***P*-trend**
	**(*n* = 792)**	**(*n* = 851)**	**(*n* = 796)**		**(*n* = 653)**	**(*n* = 639)**	**(*n* = 680)**	
DVS (median)	40	55	67		41	54	65	
Grains	461.7 ± 2.71	414.5 ± 2.44	385.0 ± 2.47	<0.0001	452.9 ± 3.31	396.0 ± 3.20	360.8 ± 3.06	<0.0001
Refined grains	179.7 ± 7.65	133.9 ± 6.06	136.0 ± 5.80	<0.0001	102.4 ± 7.21	74.3 ± 5.34	77.6 ± 4.56	0.0338
Vegetables	151.6 ± 3.48	143.9 ± 2.92	135.8 ± 2.58	0.6870	157.0 ± 4.05	139.5 ± 3.28	142.8 ± 3.04	0.6130
Fruits	65.8 ± 2.35	101.2 ± 2.80	104.9 ± 2.65	<0.0001	117.2 ± 4.17	164.4 ± 4.33	158.7 ± 3.93	0.0029
Dairy	45.3 ± 2.42	57.5 ± 2.14	60.9 ± 1.91	<0.0001	64.9 ± 3.24	82.8 ± 3.26	84.5 ± 2.58	0.0050
Meat	32.6 ± 0.96	44.2 ± 0.86	53.9 ± 0.88	<0.0001	24.9 ± 0.83	37.2 ± 0.92	48.3 ± 0.10	<0.0001
Fast foods	0.57 ± 0.15	1.06 ± 0.14	2.57 ± 0.17	<0.0001	0.79 ± 0.18	1.97 ± 0.21	3.31 ± 0.22	<0.0001
Highly palatable foods	13.7 ± 1.05	17.8 ± 0.90	24.6 ± 1.07	<0.0001	12.2 ± 1.45	13.1 ± 0.81	20.0 ± 1.04	<0.0001

DVS, dietary variety score. Values are expressed as mean (SE). The P-trend was obtained through generalized linear models after adjusting for age, BMI, marital status, monthly household income, education level, alcohol consumption, smoking status and physical activity.

### Associations between stress levels and nutrients intake

The associations of stress levels with nutrients intake per 1,000 kcal are presented in Table 4. Participants with high stress showed low total energy intake (*p* < 0.05 in men and *p* < 0.01 in women). Women with higher levels of stress showed a higher carbohydrate intake despite a lower total energy intake (*p* < 0.05). In women, there was a negative association between stress levels and most of nutrients intake (all *p* < 0.05). The intake of vitamin A, sodium, zinc, retinol, carotene, and cholesterol was not significantly differed with stress levels among women. In men, only vitamin B_1_ intake differed significantly in relation to stress levels (*p* < 0.05).

**Table 4:** Nutrient intake according to stress levels.

	**Men**				**Women**			
	**T1** **(Lowest) ^§^**	**T2** **(Intermediate)**	**T3** **(Highest)**	***P*-trend**	**T1** **(Lowest)**	**T2** **(Intermediate)**	**T3** **(Highest)**	***P*-trend**
	**(*n* = 976)**	**(*n* = 790)**	**(*n* = 673)**		**(*n* = 495)**	**(*n* = 720)**	**(*n* = 757)**	
Energy (kcal)	1,922.7 ± 17.15	1,869.0 ± 18.38	1,839.8 ± 19.39	0.0134	1,732.5 ± 23.09	1,674.4 ± 17.51	1,611.9 ± 17.78	0.0004
Protein (g)	32.7 ± 0.17	32.3 ± 0.19	32.1 ± 0.21	0.0828	33.2 ± 0.28	32.5 ± 0.20	32.1 ± 0.22	0.0161
Fat (g)	15.9 ± 0.18	15.4 ± 0.19	15.9 ± 0.22	0.8258	15.0 ± 0.26	14.4 ± 0.20	13.8 ± 0.20	0.0109
Carbohydrate (g)	178.5 ± 0.51	179.9 ± 0.53	178.7 ± 0.61	0.5906	181.3 ± 0.75	182.8 ± 0.56	184.0 ± 0.57	0.0406
Calcium (mg)	223.4 ± 2.80	218.0 ± 3.17	216.8 ± 3.54	0.2740	274.5 ± 5.28	256.2 ± 3.98	250.0 ± 3.98	0.0026
Phosphorus (mg)	494.3 ± 2.63	487.5 ± 2.90	485.4 ± 3.26	0.0872	526.8 ± 4.69	510.7 ± 3.51	504.3 ± 3.56	0.0040
Iron (mg)	5.23 ± 0.04	5.17 ± 0.05	5.07 ± 0.05	0.1125	5.82 ± 0.07	5.64 ± 0.06	5.47 ± 0.06	0.0023
Potassium (mg)	1,239.3 ± 11.65	1,226.1 ± 13.29	1,204.8 ± 13.71	0.1772	1,424.5 ± 21.77	1,366.1 ± 16.15	1,301.0 ± 16.12	0.0002
Vitamin A (R.E.)	252.8 ± 4.62	245.3 ± 5.27	246.8 ± 5.75	0.4643	278.0 ± 6.94	268.5 ± 6.03	263.6 ± 6.09	0.1737
Sodium (mg)	1,528.0 ± 24.08	1,554.1 ± 27.90	1,539.5 ± 27.85	0.9934	1,527.9 ± 35.79	1,491.7 ± 28.56	1,506.8 ± 28.22	0.5378
Vitamin B1 (mg)	0.58 ± 0.004	0.56 ± 0.004	0.56 ± 0.005	0.0197	0.56 ± 0.005	0.56 ± 0.004	0.55 ± 0.004	0.0191
Vitamin B_2_ (mg)	0.49 ± 0.004	0.48 ± 0.005	0.48 ± 0.005	0.1718	0.54 ± 0.008	0.52 ± 0.006	0.50 ± 0.006	0.0008
Niacin (mg)	7.83 ± 0.05	7.81 ± 0.06	7.75 ± 0.06	0.1049	7.90 ± 0.08	7.67 ± 0.06	7.55 ± 0.06	0.0124
Vitamin C (mg)	53.0 ± 0.81	51.2 ± 0.86	49.4 ± 0.87	0.0571	70.3 ± 1.52	67.3 ± 1.22	61.1 ± 1.11	<0.0001
Zinc (μg)	4.32 ± 0.03	4.37 ± 0.06	4.23 ± 0.03	0.4342	4.36 ± 0.04	4.28 ± 0.03	4.24 ± 0.03	0.0656
Vitamin B_6_ (mg)	0.86 ± 0.006	0.86 ± 0.007	0.85 ± 0.007	0.0926	0.92 ± 0.009	0.90 ± 0.007	0.89 ± 0.008	0.0101
Folate (μg)	114.7 ± 1.43	115.4 ± 1.71	112.4 ± 1.84	0.5526	133.5 ± 2.31	129.1 ± 1.95	126.4 ± 1.90	0.0417
Retinol (μg)	31.6 ± 0.74	29.5 ± 0.73	30.7 ± 0.86	0.5944	37.2 ± 1.19	34.0 ± 0.92	33.2 ± 0.93	0.0825
Carotene (μg)	1,285.8 ± 26.97	1,251.0 ± 30.59	1,251.7 ± 33.14	0.4552	1,408.3 ± 39.44	1,371.0 ± 35.41	1348.4 ± 34.97	0.2719
Fiber (g)	3.21 ± 0.04	3.17 ± 0.04	3.09 ± 0.04	0.1452	3.64 ± 0.05	3.55 ± 0.04	3.43 ± 0.04	0.0046
Vitamin E (mg)	4.29 ± 0.05	4.22 ± 0.05	4.21 ± 0.05	0.7252	4.88 ± 0.09	4.73 ± 0.06	4.51 ± 0.06	0.0092
Cholesterol (mg)	82.0 ± 1.61	77.5 ± 1.56	81.5 ± 2.01	0.9384	86.9 ± 2.33	81.8 ± 1.84	79.7 ± 1.99	0.1277

Values are expressed as mean (SE). Nutrient intakes were expressed per 1,000 kcal.

§ Stress levels were assessed using the Psychosocial Wellbeing Index-Short Form (PWI-SF). The P-trend was obtained through generalized linear models after adjusting for age, BMI, marital status, monthly household income, education level, alcohol consumption, smoking status and physical activity.

### Longitudinal association of food consumption with the risk of abdominal obesity

Prospectively, the highest tertile of grains and refined grains consumption showed an increased risk of abdominal obesity compared to the lowest tertile (HR: 1.36, 95% CI: 1.04–1.78, *p* < 0.05; HR: 1.28, 95% CI: 1.03–1.59, *p* < 0.05, respectively) after adjusting for all confounding factors in women (Table 5). In men, the highest tertile of refined grains consumption was associated with a higher risk of abdominal obesity compared to the lowest tertile (HR: 1.36, 95% CI: 1.11–1.66, *p* < 0.01) after adjusting for all confounding factors. By contrast, among women, the highest tertile of dairy consumption decreased the risk of abdominal obesity compared to the lowest tertile (HR: 0.79, 95% CI: 0.63–0.99, *p* < 0.05) after adjusting for all confounding factors. In all participants, the highest tertile of fruits consumption decreased the risk of abdominal obesity (men, HR: 0.56, 95% CI: 0.45–0.70, *p* < 0.01; women, HR: 0.51, 95% CI: 0.40–0.65, *p* < 0.01) after adjusting for all confounding factors.

**Table 5:** Hazard ratios (HRs) and 95% confidence intervals (CIs) for the risk of abdominal obesity according to food consumption.

	**Men**					**Women**			
	**Model 1** ^a^		**Model 2** ^b^			**Model 1**		**Model 2**	
	**HR (95% CI)**	***P*-value**	**HR (95% CI)**	***P*-value**		**HR (95% CI)**	***P*-value**	**HR (95% CI)**	***P*-value**
Grains (g/day)					Grains (g/day)				
Tertile 1 (*n* = 813)	1 (reference)	-	1 (reference)	-	Tertile 1 (*n* = 689)	1 (reference)	-	1 (reference)	-
Tertile 2 (*n* = 813)	1.154 (0.945–1.408)	0.1593	1.225 (0.991–1.514)	0.0604	Tertile 2 (*n* = 626)	1.096 (0.883–1.361)	0.4069	1.032 (0.820–1.300)	0.7865
Tertile 3 (*n* = 813)	1.094 (0.893–1.341)	0.3855	1.206 (0.919–1.583)	0.1764	Tertile 3 (*n* = 657)	1.166 (0.947–1.435)	0.1472	1.362 (1.043–1.780)	0.0233
Refined grains (g/day)					Refined grains (g/day)				
Tertile 1 (*n* = 813)	1 (reference)	-	1 (reference)	-	Tertile 1 (*n* = 657)	1 (reference)	-	1 (reference)	-
Tertile 2 (*n* = 812)	1.141 (0.932–1.397)	0.2016	1.098 (0.892–1.352)	0.3763	Tertile 2 (*n* = 658)	0.959 (0.773–1.189)	0.7011	1.031 (0.828–1.284)	0.7826
Tertile 3 (*n* = 814)	1.257 (1.031–1.533)	0.0238	1.359 (1.112–1.661)	0.0027	Tertile 3 (*n* = 657)	1.124 (0.912–1.384)	0.2739	1.282 (1.032–1.593)	0.0247
Vegetables (g/day)					Vegetables (g/day)				
Tertile 1 (*n* = 813)	1 (reference)	-	1 (reference)	-	Tertile 1 (*n* = 657)	1 (reference)	-	1 (reference)	-
Tertile 2 (*n* = 813)	0.863 (0.707–1.054)	0.1488	0.827 (0.675–1.012)	0.0656	Tertile 2 (*n* = 658)	1.052 (0.849–1.304)	0.6432	1.105 (0.889–1.373)	0.3686
Tertile 3 (*n* = 813)	0.961 (0.789–1.169)	0.6889	0.971 (0.789–1.194)	0.7774	Tertile 3 (*n* = 657)	1.156 (0.935–1.430)	0.1801	1.270 (1.016–1.589)	0.0361
Fruits (g/day)					Fruits (g/day)				
Tertile 1 (*n* = 813)	1 (reference)	-	1 (reference)	-	Tertile 1 (*n* = 657)	1 (reference)	-	1 (reference)	-
Tertile 2 (*n* = 813)	0.761 (0.626–0.926)	0.0063	0.700 (0.571–0.858)	0.0006	Tertile 2 (*n* = 658)	0.648 (0.529–0.793)	<0.0001	0.655 (0.531–0.807)	<0.0001
Tertile 3 (*n* = 813)	0.748 (0.615–0.911)	0.0038	0.564 (0.450–0.706)	<0.0001	Tertile 3 (*n* = 657)	0.501 (0.404–0.621)	<0.0001	0.513 (0.405–0.651)	<0.0001
Dairy (g/day)					Dairy (g/day)				
Tertile 1 (*n* = 816)	1 (reference)	-	1 (reference)	-	Tertile 1 (*n* = 657)	1 (reference)	-	1 (reference)	-
Tertile 2 (*n* = 811)	1.022 (0.841–1.241)	0.8282	1.007 (0.826–1.228)	0.9451	Tertile 2 (*n* = 656)	0.848 (0.690–1.043)	0.1183	0.920 (0.741–1.141)	0.4471
Tertile 3 (*n* = 812)	0.850 (0.694–1.041)	0.1153	0.817 (0.661–1.010)	0.0620	Tertile 3 (*n* = 659)	0.740 (0.599–0.915)	0.0053	0.793 (0.634–0.992)	0.0419
Meat (g/day)					Meat (g/day)				
Tertile 1 (*n* = 813)	1 (reference)	-	1 (reference)	-	Tertile 1 (*n* = 657)	1 (reference)	-	1 (reference)	-
Tertile 2 (*n* = 813)	1.000 (0.818–1.223)	0.9995	0.864 (0.698–1.070)	0.1803	Tertile 2 (*n* = 658)	0.824 (0.668–1.017)	0.0714	1.029 (0.822–1.288)	0.8030
Tertile 3 (*n* = 813)	1.078 (0.885–1.314)	0.4539	0.890 (0.700–1.132)	0.3437	Tertile 3 (*n* = 657)	0.796 (0.646–0.980)	0.0316	1.068 (0.833–1.370)	0.6040
Fast foods (g/day)					Fast foods (g/day)				
Tertile 1 (*n* = 0)	-	-	-	-	Tertile 1 (*n* = 0)	-	-	-	-
Tertile 2 (*n* = 1,739)	1 (reference)	-	1 (reference)	-	Tertile 2 (*n* = 1,318)	1 (reference)	-	1 (reference)	-
Tertile 3 (*n* = 700)	0.953 (0.797–1.138)	0.5929	0.988 (0.820–1.191)	0.9013	Tertile 3 (*n* = 654)	0.786 (0.651–0.948)	0.0121	0.903 (0.737–1.107)	0.3276
Highly palatable foods (g/day)					Highly palatable foods (g/day)				
Tertile 1 (*n* = 811)	1 (reference)	-	1 (reference)	-	Tertile 1 (*n* = 704)	1 (reference)	-	1 (reference)	-
Tertile 2 (*n* = 817)	0.953 (0.780–1.163)	0.6329	0.934 (0.762–1.143)	0.5062	Tertile 2 (*n* = 611)	1.134 (0.920–1.399)	0.2382	1.149 (0.928–1.422)	0.2017
Tertile 3 (*n* = 811)	0.993 (0.814–1.212)	0.9464	1.128 (0.906–1.404)	0.2811	Tertile 3 (*n* = 657)	0.960 (0.777–1.186)	0.7044	1.092 (0.872–1.369)	0.4421

HR, hazard ratio; CI, confidence interval; Ref., reference category.

a Model 1 was unadjusted.

b Model 2 was adjusted for age, BMI, marital status, monthly household income, education level, alcohol consumption, and total energy intake.

### Longitudinal association of stress with the risk of abdominal obesity

High stress showed a borderline significant association with the risk of abdominal obesity (HR: 1.27, 95% CI: 1.00–1.59, *p* < 0.05) after adjusting for age, BMI, marital status, monthly household income, education level, alcohol consumption, smoking status, and physical activity only in women (Table 6).

**Table 6:** Hazard ratios (HRs) and 95% confidence intervals (CIs) for the risk of abdominal obesity according to stress levels.

		**Men**						**Women**			
		**Model 1^a^**		**Model 2^b^**				**Model 1**		**Model 2**	
		**HR (95% CI)**	***P*-value**	**HR (95% CI)**	***P*-value**			**HR (95% CI)**	***P*-value**	**HR (95% CI)**	***P*-value**
Stress levels ^§^	Number of cases					Stress levels	Number of cases				
Tertile 1 (*n* = 976)	294 (30.1)	1 (reference)	-	1 (reference)	-	Tertile 1 (*n* = 495)	146 (29.4)	1 (reference)	-	1 (reference)	-
Tertile 2 (*n* = 790)	209 (26.4)	0.849 (0.701–1.028)	0.0941	0.977 (0.804–1.186)	0.8109	Tertile 2 (*n* = 720)	230 (31.9)	1.212 (0.966–1.520)	0.0960	1.324 (1.053–1.664)	0.0163
Tertile 3 (*n* = 673)	188 (27.9)	0.876 (0.717–1.071)	0.1979	1.106 (0.898–1.362)	0.3424	Tertile 3 (*n* = 757)	247 (32.6)	1.154 (0.918–1.451)	0.2206	1.265 (1.003–1.594)	0.0470

HR, hazard ratio; CI, confidence interval; Ref., reference category.

§ Stress levels were assessed using the Psychosocial Wellbeing Index-Short Form (PWI-SF).

a Model 1 was unadjusted.

b Model 2 was adjusted for age, BMI, marital status, monthly household income, education level, alcohol consumption, smoking status and physical activity.

## Discussion

In this prospective cohort study, we found that higher levels of stress affected eating behavior represented by a low DVS, characterized by higher consumption of grains and refined grains, and a lower consumption of fruits only in women. High consumption of grains, especially refined grains, was longitudinally associated with an increased risk of abdominal obesity. In addition, stress levels were positively associated with the risk of abdominal obesity. To the best of our knowledge, this is the first study to examine the associations of perceived stress, eating behavior, and abdominal obesity in Korean adults.

Stress can be defined as the generalized, non-specific response of the body to a real or perceived threat beyond the ability to cope (9). The PWI-SF, a survey used in our study, has been widely adopted to assess the levels of psychosocial stress, including physical and psychological symptoms (27, 34) in different populations (35–37). Chronic psychosocial stress is known to increase the risk of developing numerous diseases, such as metabolic syndrome (38), diabetes mellitus (39), and obesity (8).

In this study, participants who were less educated, had a lower income, and were current smokers reported higher levels of stress. Previous studies have reported that the responses to stress may influence lifestyle behaviors, such as smoking, physical activity, and alcohol use (14, 40). Cohort studies in Finland found that work stress was positively associated with both smoking status and intensity (41). Our findings are consistent with previous studies that linked lower incomes and education levels with higher levels of stress (42, 43).

Several studies have found associations between stress and unhealthy eating behavior (3, 44, 45). Eating behavior was commonly assessed based on food preferences, dietary intake, dietary variety, and eating traits (46). It is known that stressful conditions lead to a decreased dietary variety as people tend to show an increased preference for comfort foods from the same food category under stressful conditions (47, 48). In our study, women with higher levels of stress showed a lower DVS, suggesting that stress might be related to unhealthy eating behavior. Exposure to chronic stress activates the hypothalamic–pituitary–adrenal axis, with the release of cortisol (9). Increased levels of cortisol in response to stress may affect appetite (47) and promote abnormal eating behaviors (49), including preferentially selecting highly palatable foods and energy-dense foods (50, 51). The consumption of energy-dense foods has been associated with high intakes of refined grains, processed foods, and added sugars and fats, but low intakes of fruits, vegetables, and whole grains (52, 53). We found that higher levels of stress were associated with higher consumption of grains, especially refined grains in women, but a lower consumption of fruits in both men and women. Moreover, a low DVS was positively associated with the consumption of grains and refined grains but was negatively associated with the consumption of fruits. These results concur with prior studies that lower dietary variety is associated with higher consumption of refined grains (30) and lower consumption of fruits and vegetables (54). Chronic stress may modify eating behaviors, specifically the type of foods chosen, resulting in an increased consumption of refined grains.

Increased consumption of grains, especially refined grains, was longitudinally associated with an increased risk of incident abdominal obesity in women, with a mean WC increase of 3.8 ± 0.2 cm. According to the Framingham Offspring cohort study, the frequent consumption of refined grains (≥4 servings/day) was linked to a greater mean increase in WC than infrequent consumption (<2 servings/day) during 4 years (55). A cross-sectional study conducted among Indian adults suggested that higher consumption of refined grains was significantly associated with a higher WC after adjustment for confounding factors, such as age, sex, BMI, metabolic equivalent, total energy intake, and other dietary factors (56). Another cross-sectional study showed that individuals with higher scores in the “Traditional-carbohydrate” dietary pattern, characterized by higher consumption of refined grains, potatoes, sugar, and sweets, had a 55% higher prevalence of abdominal obesity (57).

Several potential mechanisms have been suggested to explain the association between the consumption of refined grains and the risk of abdominal obesity. Refined-grain foods tend to be quickly digested (58) and have a relatively high glycemic index (GI) compared with whole-grain foods, non-starchy vegetables, legumes, and fruits (59). A high-GI diet may increase hunger and lead to overeating, resulting in excess weight gain (60). A previous study of Iranian adults linked a higher dietary GI with an increased risk of abdominal obesity (61). In experimental animals fed a high-refined carbohydrate diet, the serotonin pathway was altered, accompanied by increased expression of the serotonin transporter (*Sert*), which possibly alters satiety and hunger signals, ultimately driving abdominal obesity (62). We found that women with higher levels of stress showed a higher carbohydrate intake but a relatively low intake of other nutrients. It can be suggested that those with higher levels of stress ate more refined grains and carbohydrates, partially contributing to a higher risk of abdominal obesity after 10 years.

In our study, higher levels of stress were longitudinally associated with an increased risk of abdominal obesity in women only, not men. In a prospective cohort study in the United Kingdom, job strain, a form of psychosocial stress in the workplace, was related to an increased risk of abdominal obesity (63). Moreover, a longitudinal study on stress and metabolic syndrome found a significant positive association between the number of stressful life events and WC (23). Cortisol secretion due to stress exposure might contribute to the accumulation of abdominal fat mass (9, 64). An elevated hair cortisol concentration is positively associated with BMI and WC (65). Regarding gender, there is a difference in the stress response exhibited by men and women (66). Women have more daily stress from performing routine duties (67) and find themselves in stressful circumstances more often than men (68, 69). In addition, the stress coping styles of women are more emotion-focused compared to men, resulting in increased susceptibility to negative health consequences among women (70).

We found that increased consumption of fruits, containing a lot of antioxidant nutrients and fiber, was longitudinally associated with a decreased risk of incident abdominal obesity in both men and women. High intake of vitamin C, abundant in fruits, was reported to decrease the risk of abdominal obesity in Korean women (71). Also, a major antioxidant nutrient, vitamin E supplementation reduced visceral fat deposition in mice fed a high-fat diet through reduction in the fibrotic process, which is related to adipocyte growth and lipid accumulation (72). Dietary fiber intake has been showed reduced prevalence of abdominal obesity and negative association with WC in diabetic patients (73).

This study has several strengths. It is the first to investigate the associations of perceived stress, eating behavior, and abdominal obesity in Korean adults in a prospective study with long follow-up. Furthermore, our analysis is distinct from other prior studies of eating behavior as it applied the DVS, a novel approach, to assess eating behavior. However, there are some limitations to this study. First, we assessed food consumption only at baseline and did not determine whether the dietary patterns of participants had changed throughout the follow-up. Second, blood analysis was not performed, which could reflect changes in hormones associated with stress and appetite control.

## Conclusion

In conclusion, perceived psychosocial stress was associated with an unhealthy eating behavior represented by a low DVS, characterized by high consumption of grains, especially refined grains, and relatively low consumption of fruits in women. There was a positive, longitudinal association of stress, as well as grains consumption, with the risk of abdominal obesity. Therefore, it can be suggested that stress-modified eating behavior may be one factor contributing to the risk of abdominal obesity during the follow-up.

## Data availability statement

The original contributions presented in the study are included in the article/Supplementary material, further inquiries can be directed to the corresponding author.

## Ethics statement

The Ansung-Ansan study protocol was reviewed and approved by the Institutional Review Board of the Korea Centers for Disease Control and Prevention, and all study participants submitted written informed consent. The study was approved by the Institutional Review Board of Ewha Womans University (2021-0316, October 2021).

## Author contributions

MK and YK contributed to the conceptualization, design of the research, data analysis, writing the manuscript, and editing. All authors read and approved the final manuscript.

## Funding

This research was supported by the BK21 FOUR (Fostering Outstanding Universities for Research) funded by the Ministry of Education (MOE, Korea) and National Research Foundation of Korea (NRF-5199990614253).

## Conflict of interest

The authors declare that the research was conducted in the absence of any commercial or financial relationships that could be construed as a potential conflict of interest.

## Publisher's note

All claims expressed in this article are solely those of the authors and do not necessarily represent those of their affiliated organizations, or those of the publisher, the editors and the reviewers. Any product that may be evaluated in this article, or claim that may be made by its manufacturer, is not guaranteed or endorsed by the publisher.
