# Supplementary material for: Psychosocial stress accompanied by an unhealthy eating behavior is associated with abdominal obesity in Korean adults: A community-based prospective cohort study
Source: Front Nutr. 2022 Sep 30;9:949012. doi: 10.3389/fnut.2022.949012 (PMC9561362; doi:10.3389/fnut.2022.949012)
Supplement: Supplementary file 1 [file Table_1.DOCX]

Supplementary Table 1. Classification of food items.

| **Food group Food items** | |
| --- | --- |
| Grains | white rice, rice with beans, rice with other cereals, half-and-half rice with beans, half-and-half rice with other cereals, ramyon, wheat noodles, jajangmyeon/jjamppong, naengmyeon/buckwheat noodles, dumplings/dumpling soup, white rice cake/rice-cake soup, rice cake, corn flakes, bread/sandwich/toast, red bean bread/steamed bun/pulppang, other bread, cake/chocolate pie, pizza/hamburger, powder of roast grain |
| Refined grains | white rice, ramyon, wheat noodles, jajangmyeon/jjamppong, naengmyeon/buckwheat noodles, white rice cake/rice-cake soup, rice cake |
| Vegetables | cabbage kimchi, radish kimchi, radish water kimchi, other kimchi, pickled vegetables, radish, cabbage/cabbage soup, spinach, lettuce, sesame leaf, mixed vegetable salad, other green vegetables, deodeok/balloon flower, bean sprouts, bracken fern/sweet potato vines/taro stem, oyster mushroom, other mushrooms, pepper leaves/seasoned vegetables, crown daisy/chives/parsley, cucumber, carrot/carrot juice, onion, green pepper, young pumpkin, pumpkin/ pumpkin juice |
| Fruits | strawberry, melon, watermelon, peach/plum, banana, persimmon, tangerine, pear/pear juice, apple/apple juice, orange/orange juice/ grape/grape juice, tomato/tomato juice |
| Dairy | milk, yogurt, ice cream, cheese, cream |
| Meat | grilled pork belly, roasted pork/seasoned pork, steamed pork, processed meat, meat products, steak/roasted beef, dog meat, chicken, meat stew, sashimi, blue-backed fish, hairtail, eel, croaker/snapper/flatfish, pollack, squid/small octopus, anchovy, canned tuna, salted seafood, clam/sea snail, oyster, crab, shrimp, fish cake |
| Fast foods | pizza/hamburger, processed meat |
| Highly palatable foods | jam/honey/butter/margarine, cake/chocolate pie, cookie/cracker/snack, candy/chocolate, ice cream, sugar, soft beverage |
